# RRAD expression in gastric and colorectal cancer with peritoneal carcinomatosis

**DOI:** 10.1038/s41598-019-55767-7

**Published:** 2019-12-19

**Authors:** Hee Kyung Kim, Inkyoung Lee, Seung Tae Kim, Jeeyun Lee, Kyoung-Mee Kim, Joon Oh Park, Won Ki Kang

**Affiliations:** 1 Division of Hematology-Oncology, Departments of Internal Medicine, Samsung Medical Center, Sungkyunkwan University School of Medicine, Seoul, Korea; 2 Biological Research Institute, Samsung Medical Center, Sungkyunkwan University School of Medicine, Seoul, Korea; 3 Department of Pathology, Samsung Medical Center, Sungkyunkwan University School of Medicine, Seoul, Korea; 4 Department of Internal Medicine, Chungbuk National University Hospital, Chungbuk National University College of Medicine, Cheongju, Korea

**Keywords:** Colon cancer, Rectal cancer, Gastric cancer, Tumour biomarkers

## Abstract

The role of Ras-related associated with diabetes (RRAD) in gastric cancer (GC) or colorectal cancer (CRC) has not been investigated. We aimed to investigate the biological and clinical roles of RRAD in GC and CRC and to assess RRAD as a therapeutic target. A total of 31 cancer cell lines (17 GC cell lines, 14 CRC cell lines), 59 patient-derived cells (PDCs from 48 GC patients and 11 CRC patients), and 84 matched pairs of primary cancer tissue and non-tumor tissue were used to evaluate the role of RRAD *in vitro* and *in vivo*. RRAD expression was frequently increased in GC and CRC cell lines, and siRNA/shRNA-mediated RRAD inhibition induced significant decline of tumor cell proliferation both *in vitro* and *in vivo*. A synergistic effect of RRAD inhibition was generated by combined treatment with chemotherapy. Notably, RRAD expression was markedly increased in PDCs, and RRAD inhibition suppressed PDC proliferation. RRAD inhibition also resulted in reduced cell invasion, decreased expression of EMT markers, and decreased angiogenesis and levels of associated proteins including VEGF and ANGP2. Our study suggests that RRAD could be a novel therapeutic target for treatment of GC and CRC, especially in patients with peritoneal seeding.

## Introduction

Gastric cancer (GC) and colorectal cancer (CRC) are the most common gastrointestinal malignancies worldwide^1^. Despite declining incidence and advances in treatment, the prognosis of metastatic GC is poor, and GC is the second leading cause of cancer mortality^2,3^. Similarly, CRC is the third leading cause of cancer-related death despite the development of target therapies^4^. Advances in the genomic landscape in GC and CRC have spurred the application of target therapies. There have been numerous attempts to extrapolate clinical benefits from preclinical investigations^5–7^. Studies targeting metabolic enzymes have increased because cancer metabolism has a close relationship with genomic alterations^8^.

RRAD (Ras-Related Associated With Diabetes) is a member of the family of Ras-related GTPases, which are overexpressed in type II diabetic muscle compared with muscle of individuals that are nondiabetic or who have type I diabetes^9^. RRAD, a 35-kDa protein, is encoded by a gene located on chromosome 16q22 and is normally expressed in the heart, lung, and skeletal muscles^10^. The Ras-related GTPases are broadly involved in cellular function, including cell proliferation and differentiation^11^. Compared with other Ras-related GTPases, RRAD has a distinct function as a negative regulator of glucose uptake and also has a role in cytoskeletal organization^10^. Overexpression of RRAD in cultured muscle and adipocyte cells decreased insulin-stimulated glucose uptake^10^.

RRAD is also expressed in some malignant tumors such as breast cancer, leukemia, lymphoma, and glioma^9,12–14^. Overexpression of RRAD in breast cancer is associated with invasiveness and poor prognosis^13^. RRAD knockdown induced mitochondrial apoptosis in leukemia and lymphoma cell lines^12^. Previously, our group showed that RRAD knockdown could suppress tumor growth in prostate, breast, and stomach cell lines^15^. However, there has been little investigation of RRAD in GC or CRC, and the role of RRAD in GC remains unclear. In this study, we aimed to evaluate the biological role of RRAD in GC and CRC and to assess its potential as a therapeutic target.

## Results

### RRAD are expressed in GC and CRC cell lines with distinct levels, and RRAD knockdown reduced cell proliferation

**Figure 1:**
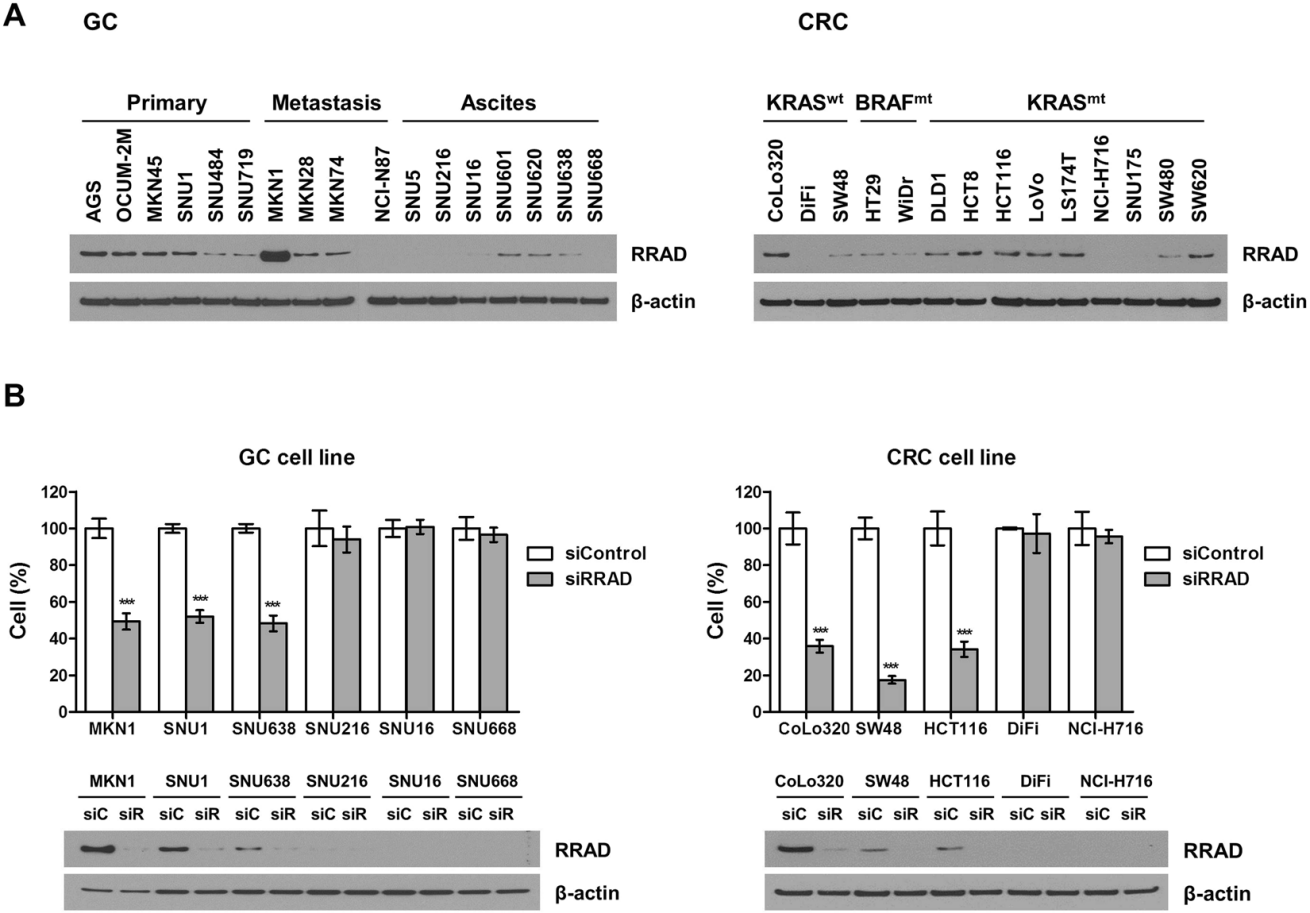
Effect of RRAD inhibition on cell proliferation. (**A**) Expression pattern of RRAD in gastric cancer and colorectal cancer cell lines. Western blot analysis was used to assess RRAD expression in 16 gastric cell lines (left, primary, N = 6; metastasis, N = 4; ascites, N = 6) and 14 CRC cell lines (right, KRAS wild type, N = 3; BRAF mutant, N = 2; KRAS mutant, N = 9). β-Actin was used as loading control. Full-length blots are presented in Supplementary Fig. S7. (**B**) RRAD knockdown by transfection of RRAD siRNA suppressed GC and CRC cell proliferation. Cell proliferation was measured 72 hours after transfection with siRRAD#1(10 nM) or negative control sequence (siC). Percentage of viable cells is shown relative to that of untreated control. Proliferation of RRAD-positive cell lines (MKN, SNU1, SNU638, CoLo320, SW48, and HCT116) was significantly inhibited by RRAD targeting siRNA, whereas proliferation of RRAD-negative cell lines (SNU216, SNU484, SNU668, DiFi, and NCI-H716) was not affected.). Full-length blots are presented in Supplementary Fig. S7. *P < 0.05, **P < 0.01, ***P < 0.001.

RRAD protein expression was evaluated using western blot in 17 GC cell lines and 14 CRC cell lines (Fig. 1A). RRAD expression was more frequently observed in CRC cell lines than GC cell lines. Among CRC cell lines, all *BRAF*-mutant CRC cell lines showed RRAD expression. RRAD-positive (strong expression by western blot) and RRAD-negative (low or no expression) cell lines were selected, and RRAD-positive cell lines were transfected with siRNA to suppress RRAD. Figure 1B shows that cell proliferation was significantly decreased by siRRAD after 72 hours in RRAD-positive cell lines. Protein expression was also suppressed by transfection with siRRAD in the same cell lines. Multiple siRRADs and shRRADs are used in the experiments and the results are shown in Fig. S1. When we compared the growth rate of RRAD-negatvie cell lines (SNU668, DiFi) and RRAD-positive cell lines (MKN1, CoLo320), RRAD-positive cell lines showed significant higher growth rate (Fig. S2).

### RRAD inhibition synergistically enhances cell death induced by chemotherapy

**Figure 2:**
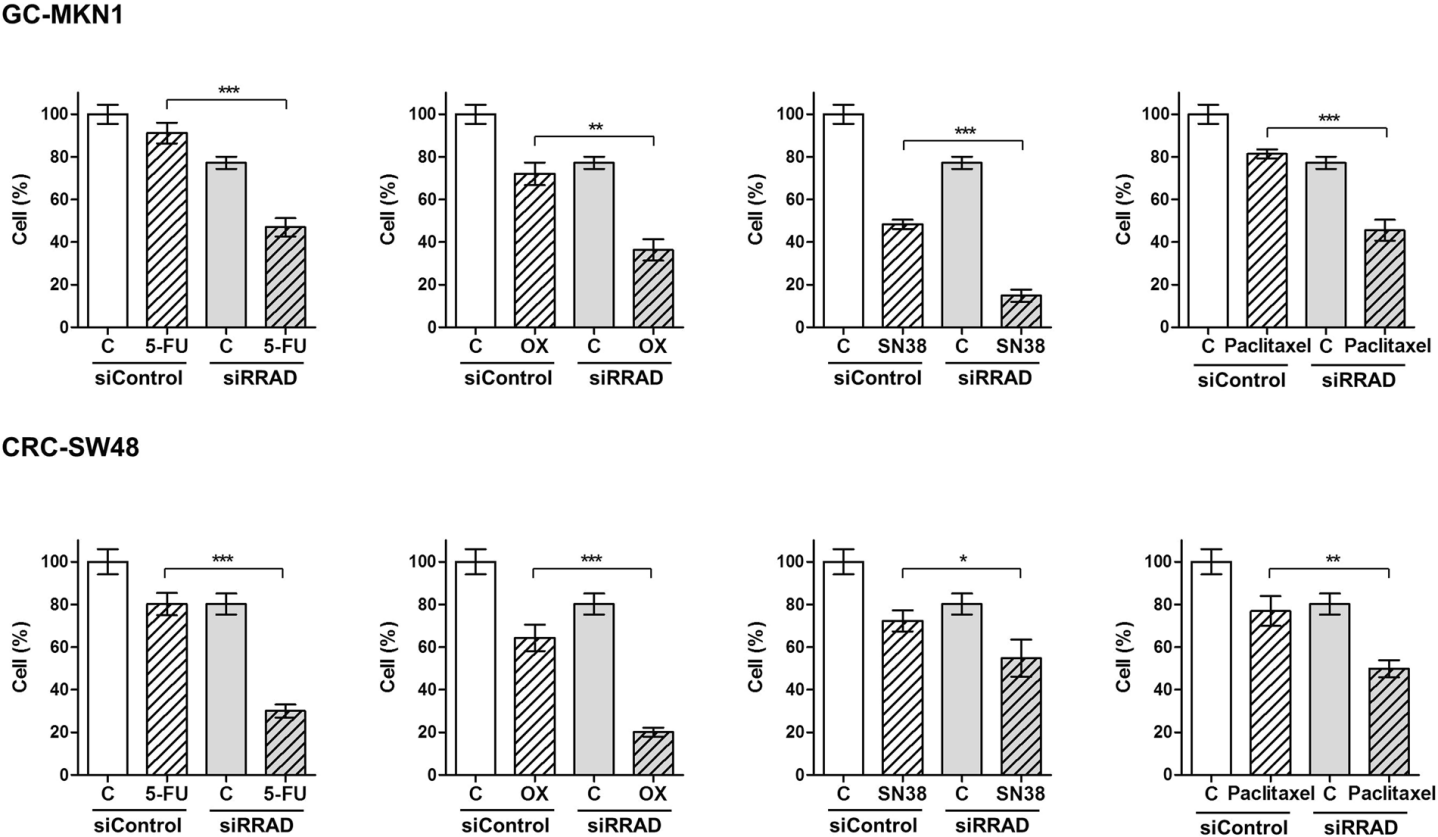
Downregulation of RRAD enhances chemo-drug cytotoxic effect. MKN1 cells and SW48 were transfected with siRRAD#1 (3 nM). The next day, cells were treated with chemo-drug for 3 days and stained with 0.4% trypan blue. Cell count is expressed as percentage of cell proliferation using the control as reference. *P < 0.05, **P < 0.01, ***P < 0.001.

Figure 2 demonstrates the synergistic effect of RRAD inhibition with chemotherapy. The MKN1 GC cell line and SW48 CRC cell line were transfected with siRRAD. Then, the cells were treated with chemotherapeutic drugs 5-fluorouracil (5-FU), oxaliplatin, SN38, and paclitaxel. Knockdown of RRAD significantly induced cell death with chemotherapeutic drug treatment. The synergism of RRAD inhibition and chemotherapy was also observed in other GC cell lines (SNU1, SNU638) and CRC cell lines (CoLo 320, HCT116) (Fig. S3). Among the four chemotherapeutic agents, 5-FU or oxaliplatin was a more effective combination with RRAD down-regulation. There was a lower proportion of cell death with combination treatment in GC cell lines compared to CRC cell lines. The synergistic effects of 5-FU and RRAD inhibition was also evaluated with detection of apoptosis using annexin V staining assay. The cell death was increased after knockdown of RRAD in the MKN1 GC cell line and SW48 CRC (Fig. S4).

### RRAD expression level differed according to tumor tissue type of normal tissue, tumor tissue, malignant ascites

**Figure 3:**
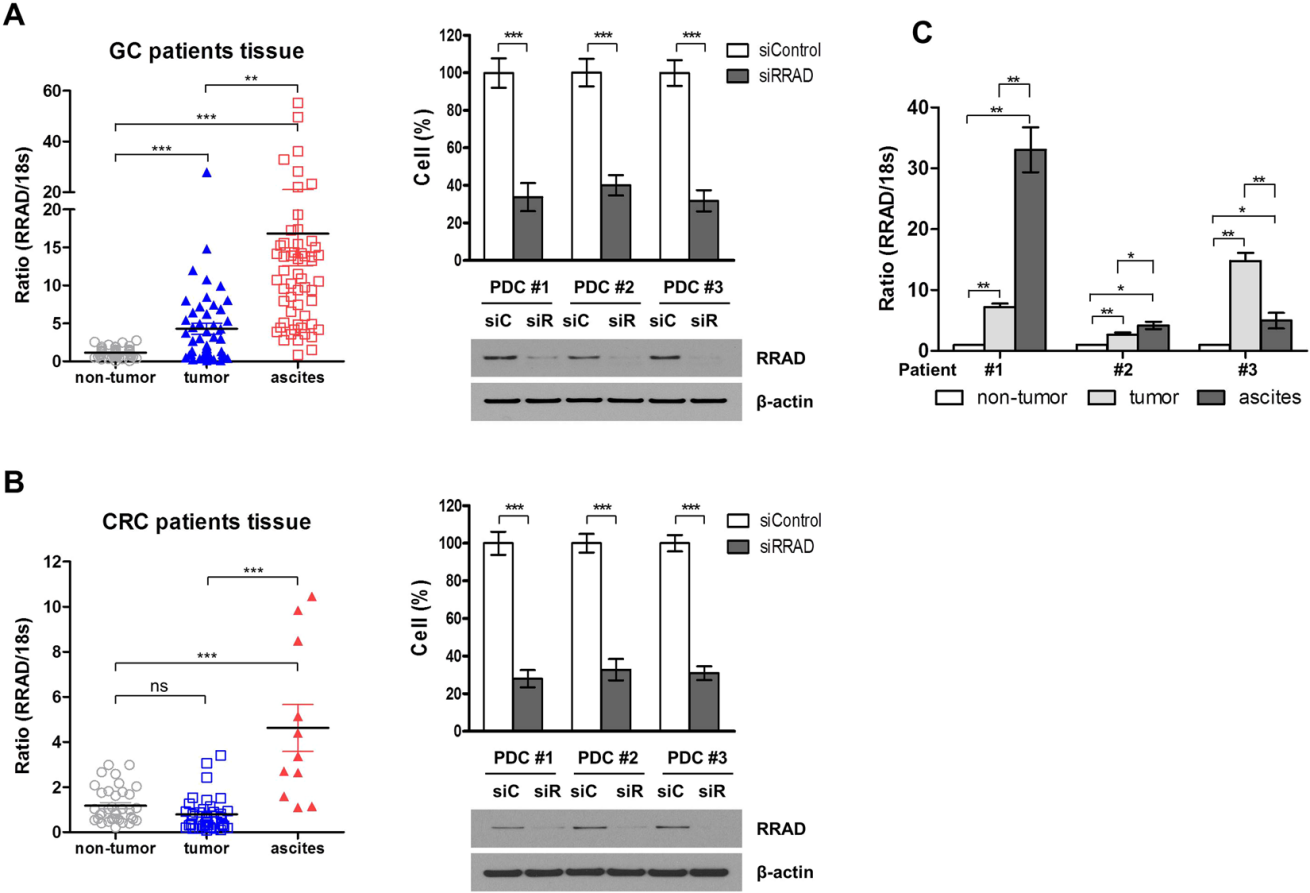
RRAD expression in tumor tissues. RRAD protein expression in gastric cancer tissues (**A**) and colorectal cancer tissues (**B**). Left, RRAD mRNA and 18 S rRNA were detected using real-time PCR according to tissue (non-tumor tissue, tumor tissue, and PDCs collected from malignant ascites). Data were normalized to 18 S rRNA as an endogenous control. Right, RRAD expression in PDCs collected from malignant ascites and effects of RRAD inhibition on cell proliferation. (**C**) Three sets of RRAD expression in GC patient-matched tissue. Full-length blots are presented in Supplementary Fig. S7. *P < 0.05, **P < 0.01, ***P < 0.001.

The RRAD expression in GC and CRC was measured by RT-PCR and compared among non-tumor tissue, tumor tissue, and PDC (patient-derived cell) from malignant ascites. In GC tissues, RRAD expression was significantly elevated in tumor and ascites compared to non-tumor tissue, but there was no significant difference in RRAD expression between tumor tissue and non-tumor tissue in CRC (Fig. 3A,B, Left). RRAD was markedly overexpressed in PDC from malignant ascites of CRC and GC compared to non-tumor tissues or primary tumor tissues. Next, the impact of RRAD knockdown in PDC was assessed by transfection with siRNA. Down-regulation of RRAD significantly decreased the cell proliferation of PDC in both CRC and GC (Fig. 3A,B, Right). RRAD expression was also examined in patient-matched samples, and all tumor tissue, including PDC from ascites, harbored more RRAD overexpression than non-tumor tissue (Fig. 3C).

### RRAD knockdown inhibits CRC and GC tumor growth *in vivo*

**Figure 4:**
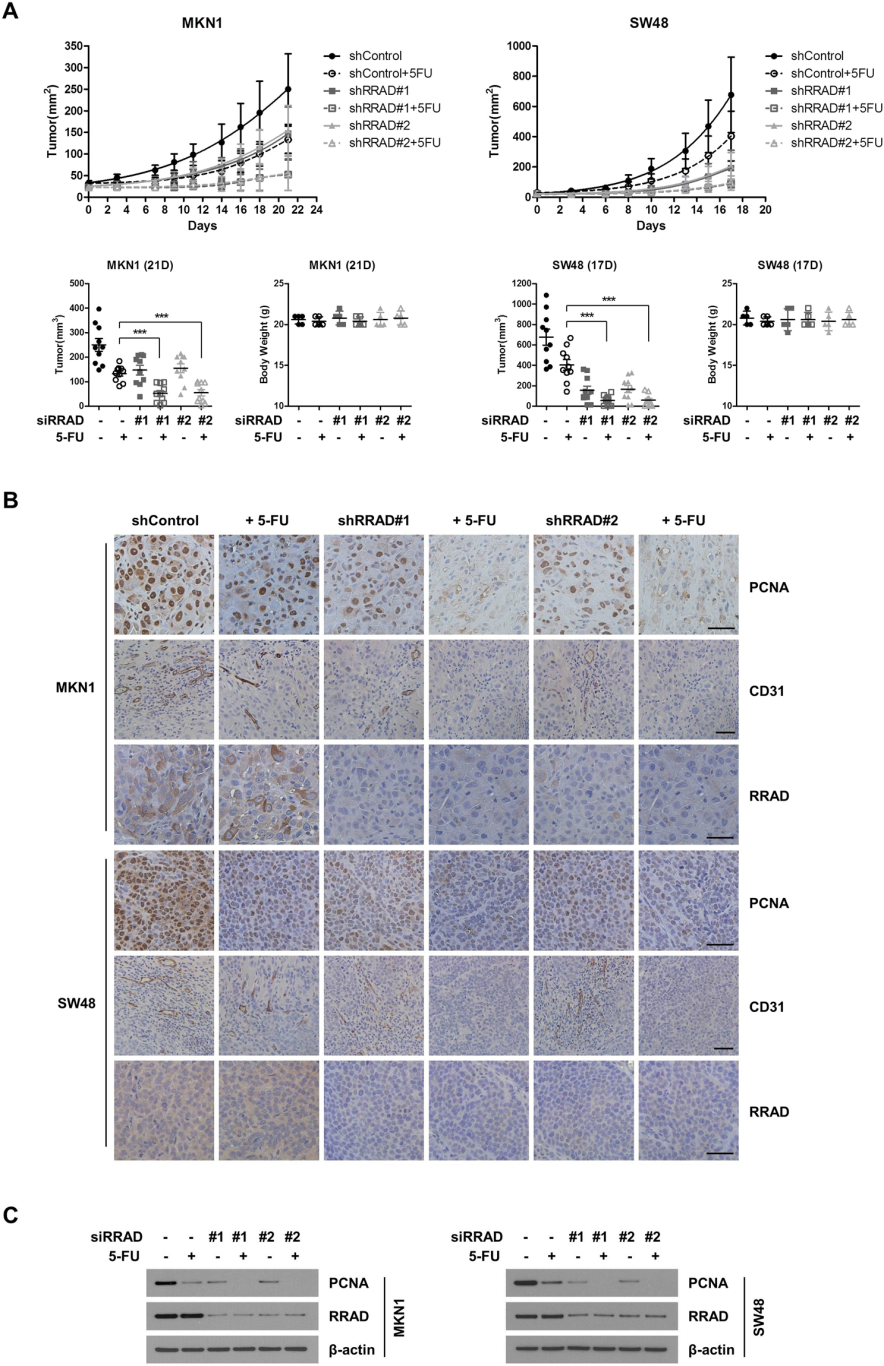
RRAD expression correlates with tumorigenesis. (**A**) RRAD knockdown decreases *in vivo* tumorigenesis. BALB/c nude mice were subcutaneously injected in bilateral flanks (2 injections per mouse) with shRRAD expressed MKN1 cells (1 × 10^7^ cells) or SW48 cells (5 × 10^6^ cells). At 7 days after inoculation, 5-FU treatment was started. 5-FU (1 mg/kg, intraperitoneal injection) were given twice per week. Upper panels show the time course of growth, and lower panels represent mean tumor volume and standard deviation. *P < 0.05, **P < 0.01, ***P < 0.001. (**B**) Immunohistochemistry staining of mouse xenograft tumors for for PCNA, CD31 and RRAD (x200, Scale bar 50 μm). (**C**) RRAD knockdown inhibits tumor growth and sensitizes to 5-FU. Level of PCNA and RRAD protein was determined by immunoblotting. Full-length blots are presented in Supplementary Fig. S7.

*In vitro* analysis could not reflect the interaction between tumor cells and tumor microenvironment, so mice bearing tumors derived from GC cells and CRC cells were treated to determine the anti-tumor effect of RRAD inhibition *in vivo* (Fig. 4). MKN1 was selected as an RRAD-positive GC cell line, and SW48 was selected as an RRAD-positive CRC cell line. MKN1 cells and SW48 cells were implanted into mice. Four groups were created according to treatment: untreated control, 5-FU, shRRAD, and combination 5-FU and RRAD. Combination 5-FU and RRAD generated the most significant decrease of MKN1 and SW48 tumor volume on days 17 and 21, respectively (Fig. 4A). A single treatment with 5-FU or shRRAD also induced significant reduction of GC and CRC tumor, and the reduced tumor volume was more apparent in SW48 CRC tumors.

For each retrieved tumor sample of xenograft, protein expression was evaluated using immunohistochemistry (IHC) with a monoclonal anti-PCNA antibody, CD31 to validate tumor growth inhibition and angiogenesis with 5-FU and shRRAD in xenografts (Fig. 4B). The PCNA, CD31 and RRAD signals of xenografts were markedly reduced when mice were treated with a combination of 5-FU and shRRAD. Quantification of CD31-positive pixels was shown in Fig. S5, is significantly reduced after treatment with a combination of 5-FU and siRRAD. Figure 4C depicts protein expression by western blot, which had similar results to IHC.

### RRAD expression is correlated with cell invasion, migration, and angiogenesis

**Figure 5:**
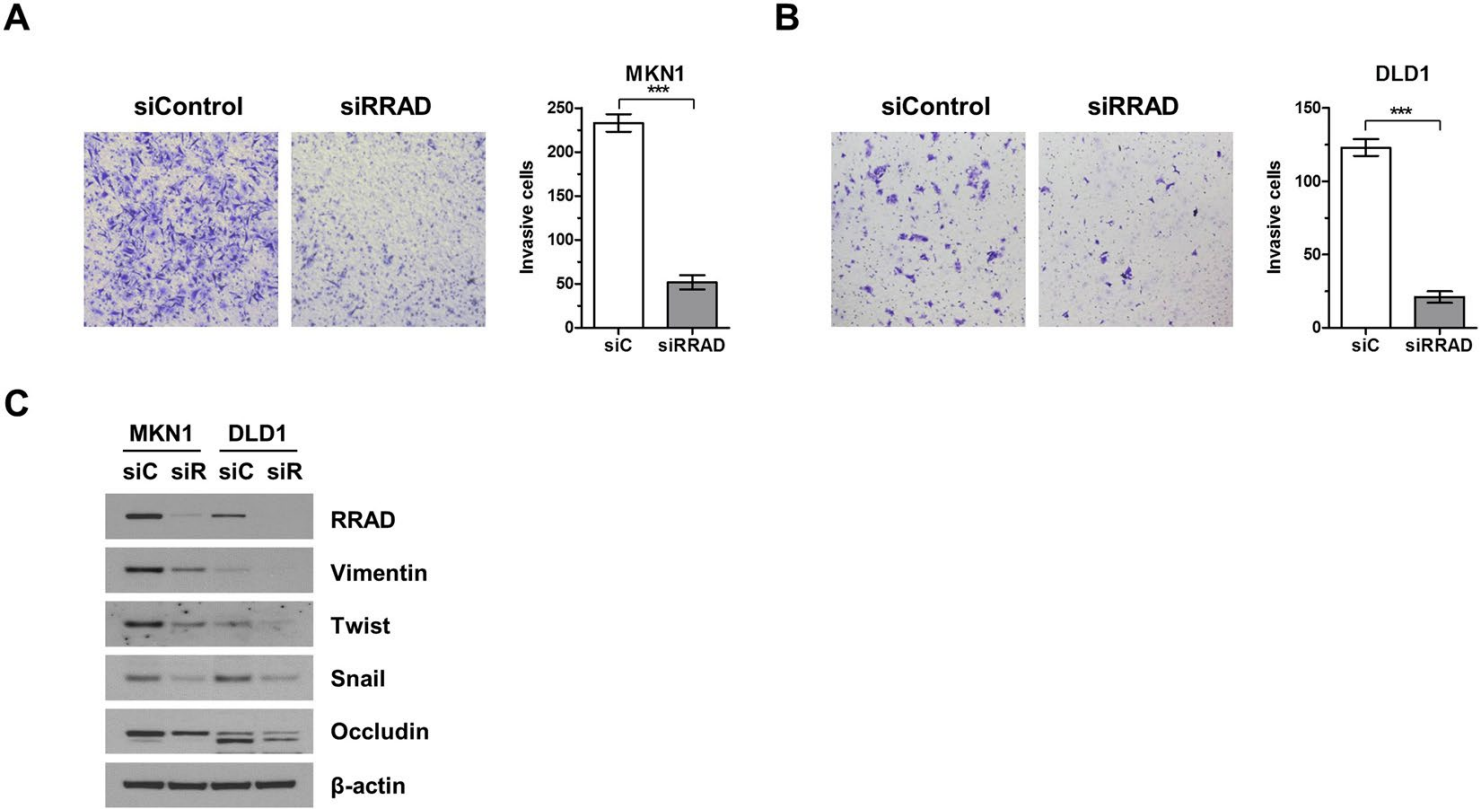
Depletion of RRAD decreases EMT-regulating gene expression. Cancer cell invasion in siRRAD#1-transfected MKN1 cells (**A**) and DLD1 cells (**B**). Cells that invaded through the membrane were stained with crystal violet and counted directly under a microscope. Data represent mean ± SD of three independent experiments. The EMT markers vimentin, twist, snail, and occludin also decreased with siRRAD by immunoblotting (**C**). Full-length blots are presented in Supplementary Fig. S8. *P < 0.05, **P < 0.01, ***P < 0.001.

To investigate whether RRAD affected cell invasion ability in GC and CRC, a modified Boyden chamber cell invasion assay was performed. First, MKN1 was selected as the GC cell line, and DLD1 was selected as the CRC cell line, both of which expressed RRAD protein. As shown in Fig. 5A,B, RRAD suppression significantly inhibited invasion of MKN1 and DLD1 cells (p < 0.001). Next, EMT (epithelial-mesenchymal transition) markers were analyzed using an immunoblot assay after transfection with siRRAD. EMT markers are known to contribute to cancer progression and metastasis^16,17^. EMT markers consisted of vimentin, twist, snail, and occludin. In the immunoblot assay, all EMT-association proteins decreased with siRRAD transfection (Fig. 5C).

**Figure 6:**
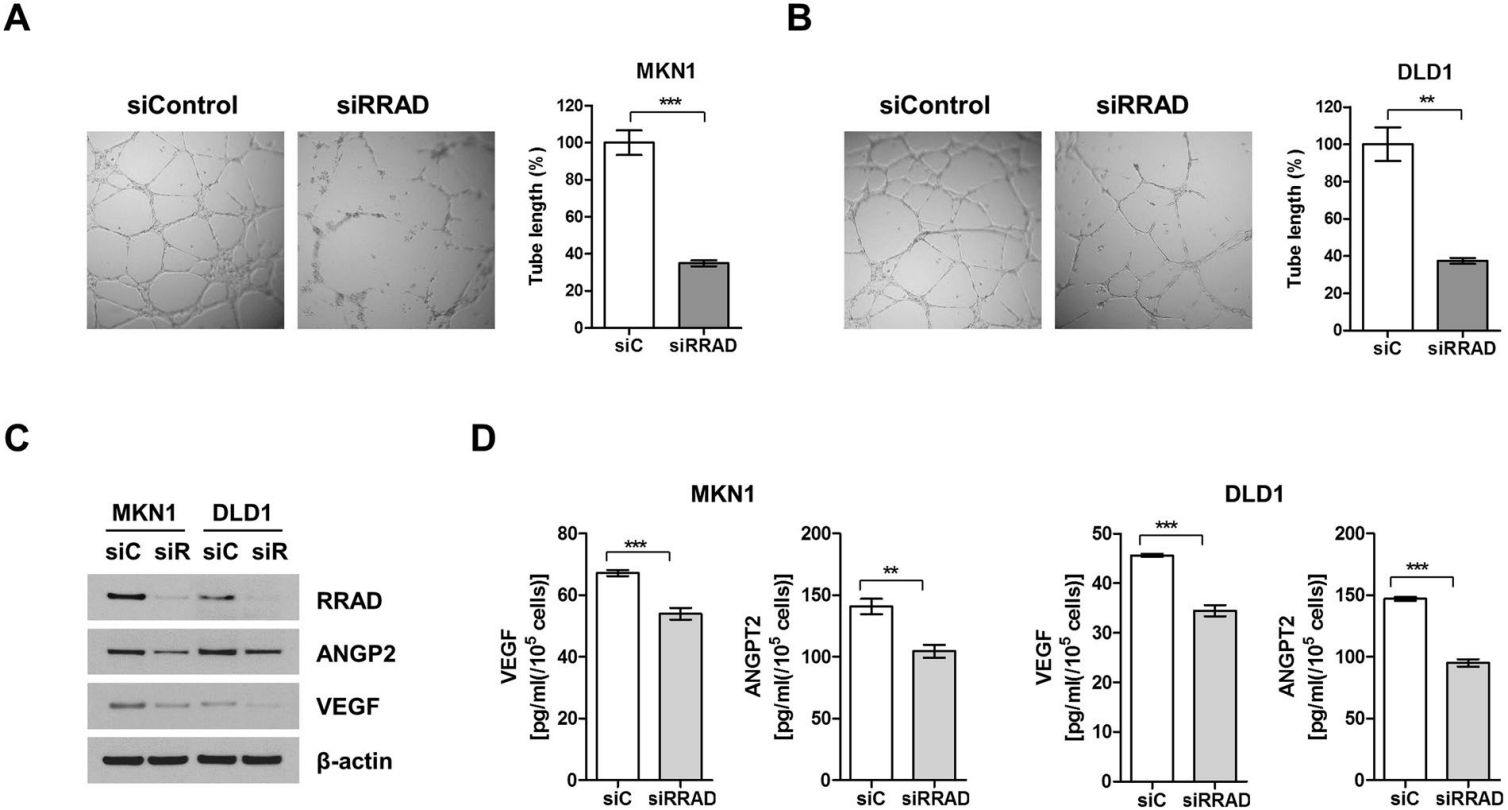
Depletion of RRAD decreases angiogenesis-related factors. HUVEC cell was seeded on Matrigel and incubated for 18 h in siControl or siRRAD#1-transfected MKN1 cells (**A**) and DLD1 cells medium (**B**). Tube formation was determined by assessment of the total length of tube in three randomly selected fields. Data represent mean ± SD of three independent experiments. Angiogenesis-related factors including VEGF and angiopoietin 2 were also decreased by siRRAD with immunoblotting (**C**) and ELISA analysis (**D**). Full-length blots are presented in Supplementary Fig. S8. *P < 0.05, **P < 0.01, ***P < 0.001.

Because cell invasion and migration are two key steps for angiogenesis and metastasis^18^, HUVEC cell tube formation in MKN1 and DLD1 cells was assessed after treatment with siRRAD. Compared with the control, significant decreases in HUVEC migration were observed in both cell lines with siRRAD (Fig. 6A,B). Next, immunoblot and ELISA were performed to analyze the correlations between RRAD expression and angiogenesis-related factors. In the immunoblot assay, VEGF and angiopoietin 2 were decreased by siRRAD **(**Fig. 6C). The result of ELISA analysis was in concordance with the result of immunoblot (Fig. 6D).

### RRAD up-regulation promotes cell proliferation and migration

**Figure 7:**
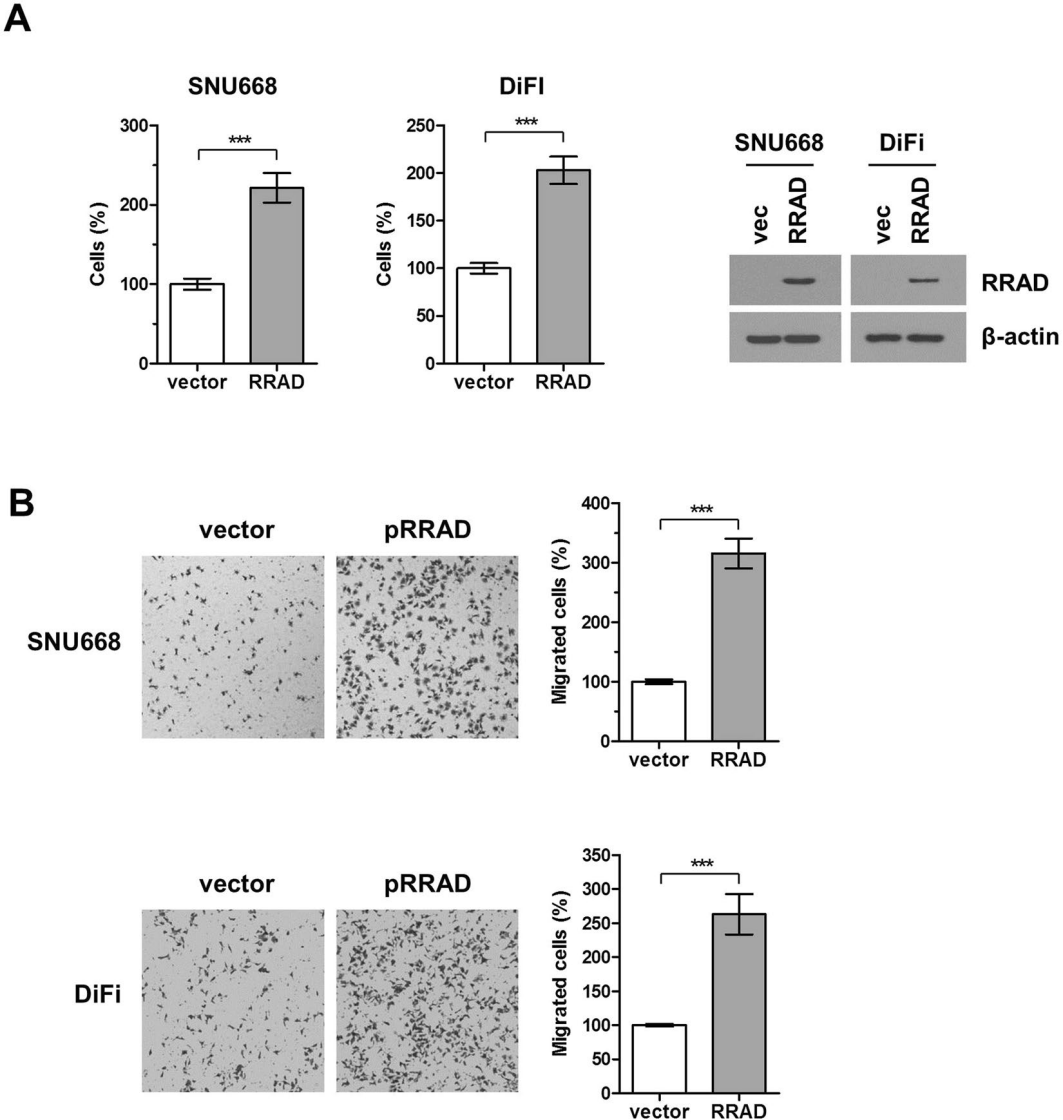
Overexpression of RRAD increased cell proliferation and migration. SNU668 and DiFi cell were transfection with RRAD or pCMVTag2B vector. (**A**) Cell proliferation was measured 72 hours after plasmid transfection. Percentage of viable cells is shown relative to that of vector control. (**B**) Cells that migrated through the membrane were stained with crystal violet and counted directly under a microscope. RRAD expression was analyzed with immunoblotting using actin as a loading control. Data represent mean ± SD of three independent experiments. Full-length blots are presented in Supplementary Fig. S8.

We next assessed the effects of RRAD overexpression and cell proliferation and migration. The proliferation of RRAD-negative CRC and GC cell lines increased following RRAD expression by transfection with RRAD plasmid. 72 hours after the transfection, cell proliferation was significantly increased in each respective cell line (SNU668 and DiFi, Fig. 7A). To ascertain whether the effect of RRAD overexpression is correlated with cell invasion and migration, we performed Boyden chamber cell invasion assay in SNU668 and DiFi cell lines. Cell migration was significantly increased after RRAD overexpression by plasmid transfection (Fig. 7B).

## Discussion

Altered expression of RRAD is frequently observed in cancer tissues, and it was associated with poor prognosis in several cancers including breast cancer, lung cancer^19^, nasopharyngeal cancer^20^, and ovarian cancer^21^. In this study, we described RRAD expression in CRC and GC, which has not yet been established. Quantitative RT-PCR assay and western blot assay showed that RRAD was markedly overexpressed in malignant cells of ascites, and RRAD inhibition resulted in suppression of cancer cell proliferation and invasion in CRC and GC cell lines. To the best of our knowledge, our report provides the first evidence that RRAD is a potential therapeutic target in GC and CRC with peritoneal carcinomatosis.

A change in glycolysis is inevitable in accordance with high oxygen tension in aggressive and invasive cancer cells^22,23^. Change in energy production from oxidative phosphorylation to aerobic glycolysis is known as the Warburg effect^24,25^. The Warburg effect promotes cancer cell growth and invasion, and correlation with RRAD in tumor tissues was recently reported^26–28^. In addition, RRAD was identified as a negative regulator of the Warburg effect and cancer progression in breast cancer, nasopharyngeal cancer, hepatocellular carcinoma, and lung cancer^19,20,27,29^. In contrast, our study showed that RRAD expression was associated with cancer invasion, migration, and angiogenesis. Furthermore, when considered RRAD could be a negative regulator of the glycolysis, KRAS mutation should have been related with RRAD. KRAS mutation is frequently observed in CRC patients and increases the glycolysis in cancer cells^30^. However, KRAS mutation was not correlated with RRAD expression in the current study. We conducted the study to evaluate the effect of RRAD inhibition on glucose uptake, but there was no difference in glucose uptake after RRAD knockdown in MKN1 and SW48 cell lines (Fig. S6A). In the lactate production assay, the level of lactate was decreased by transfection with siRRAD (Fig. S6B). These finding suggests that RRAD is a positive regulator of the aerobic glycolysis without increasing uptake of glucose in GC or CRC. To elucidate the role of RRAD in gastrointestinal cancers including GC and CRC, further analysis is needed.

RRAD inhibition had a synergistic effect with chemotherapeutic agents in treatment of GC or CRC cell lines in our study. We conducted combination therapy with RRAD inhibition and chemotherapeutic agents assuming the RRAD inhibition alone was insufficient to control GC and CRC. Chemotherapy regimens are commonly used in GC and CRC, including 5-FU, oxaliplatin, taxane, or irinotecan. Herein, we demonstrate that a combination of RRAD inhibition with chemotherapeutic agents in RRAD-expressed cell lines of GC or CRC resulted in more effective antitumor efficacy than monotherapy. Taxane (paclitaxel) showed synergism with RRAD inhibition in SW48 which was CRC cell line, although paclitaxel was not standard treatment of CRC. These results support the feasibility of RRAD inhibitor as a therapeutic target for treatment of GC and CRC.

The epithelial-mesenchymal transition (EMT) is known to be involved in cancer progression and invasion^31^, and we demonstrated that RRAD inhibition could suppress cell invasion and expression of EMT-associated proteins. In addition, we evaluated the impact of RRAD on tumor angiogenesis. RRAD inhibition could also decrease VEGF and ANGP2 in GC and CRC cell lines. VEGF is a positive regulator of tumor angiogenesis, and VEGF inhibitors are widely used in cancer treatment^32^. ANGP2 is also involved in angiogenesis of tumor tissue^33^. Invasion, migration, and angiogenesis are key factors in cancer cell progression and metastasis. Our results showed that RRAD could suppress key cellular processes in GC and CRC cell lines. However, there are still several questions that have to be answered, including the mechanism underlying RRAD expression and EMT or angiogenesis. The further studies are warranted to delineate the mechanisms of RRAD expression and cancer progression.

Among the patterns of recurrence in CRC and GC, peritoneal dissemination poses an intractable clinical problem. The prognosis of GC and CRC peritoneal carcinomatosis is dismal, and the median survival is about 6 months after diagnosis^34,35^. In our study, RRAD was prominently expressed in ascites PDC lines from GC and CRC compared to tumor and non-tumor tissues. This finding suggests the success rate of the PDC establishment and the significance of RRAD in cancer progression. In addition, RRAD inhibition synergistically increased the effects of several cytotoxic drugs that were used to treat GC and CRC. Based on these results, RRAD inhibitor could be a breakthrough in treatment of peritoneal carcinomatosis in advanced GC and CRC.

In this study, we investigated the mechanism by which RRAD expression regulated tumor invasion and progression. We also evaluated RRAD as a therapeutic target in treatment of GC and CRC. Our group has previously reported that RRAD inhibition might suppress carcinogenesis *in vitro* and *in vivo*^15^. To the best of our knowledge, this study is the first to show the anti-tumor efficacy of RRAD inhibition in GC and CRC. Our results suggest that RRAD inhibitor might be a novel strategy for treatment of GC and CRC, including patients with peritoneal carcinomatosis.

## Methods

### Cell culture

Fourteen known human CRC cell lines (CoLo320, DiFi, SW48, HT29, WiDr, DLD1, HCT8, HCT116, LoVo, LS174T, NCI-H716, SNU175, SW480, and SW620) and 17 known human GC cell lines (6 primary cell lines-AGS, OCUM-2M, MKN45, SNU1, SNU484, and SNU719, 4 metastatic cell lines-MKN1, MKN28, MKN74, and SNU216, and 7 ascites cell lines-SNU5, SNU216, SNU16, SNU601, SNU620, SNU638, and SNU668) were used. Most of these cell lines were purchased from American Type Culture Collection (Manassas, VA, USA) and the Korean Cell Line Bank (Seoul, Korea), except for the DiFi cell line that was generously provided by Dr. JO Park (Samsung Medical Center, Seoul, Korea).

To establish PDCs, malignant ascites effusions were collected from patients with metastatic cancer. Collected effusions (1–5 L) were divided into 50-mL tubes, centrifuged at 1500 rpm for 10 min, and washed twice with PBS. Cell pellets were resuspended in culture medium and plated into 75-cm^2^ culture flasks.

All cells and PDCs were grown in RPMI-1640 medium supplemented with 10% FBS, an antibiotic, and an antimycotic (Invitrogen Corporation, Carlsbad, CA, USA). Cells were cultured at 37 °C in a humidified 5% CO_2_ environment.

### Western blot analyses

Total cell extracts were obtained using lysis buffer (20 mM HEPES [pH 7.4], 1% Triton X-100, 1 mM EDTA, 1 mM MgCl_2_, 150 mM NaCl, 10% glycerol, and protease inhibitor cocktail [Invitrogen]), and protein concentration was determined using the micro-BCA protein reagent (Pierce Biotechnology, Rockford, IL, USA). Equal amounts of proteins (30 μg per well) from clarified lysates were separated by sodium dodecyl sulfate–polyacrylamide gel electrophoresis and transferred onto nitrocellulose membranes with 0.45-μm pores (Whatman, Maidstone, UK). The membranes were sequentially incubated in 5% dry milk and antibodies against RRAD (Abcam, Cambridge, U.K, ab75100, 1:1000), β-actin (Sigma, St Louis, MO, USA, A5441, 1:5,000), PCNA (Santa Cruz Biotechnology, CA, sc-56, 1:1,000), vimentin (Cell Signaling, Danvers, MA, 5741S, 1:1,000), Twist (Santa Cruz, sc-15393, 1:1,000), snail (Cell Signaling, 3879S, 1:1,000), Occludin (Cell Signaling, 5446S, 1:1000), angiopoietin 2 (AbFrontier, Seoul, Korea, LF-PA50005, 1:500), and BiP (BD Biosciences, San Jose, CA, USA, 610978, 1:1,000). The ECL system was used for protein detection (Invitrogen).

### qRT-PCR

Total cellular RNA was extracted using the RNeasy MiniKit (Qiagen, Valencia, CA) and was treated with DNase I (Qiagen). One microgram of RNA was converted to cDNA using an Omniscript RT Kit (Qiagen). Real-time PCR was performed using a Priam 7900HT Sequence Detection System (PE Applied Biosystems). RRAD mRNA and 18S rRNA were detected using TaqMan Gene Expression Master Mix Reagent and TaqMan probe (Applied Biosystems). Data were normalized to 18S rRNA as an endogenous control and were calculated using the comparative Ct method (2-delta delta Ct).

### RNA interference and transfection

The 21-nucleotide-long siRNAs corresponding to RRAD (siRRAD#1, 5′-GCAAGUUCAUUGAGACAUCUU-3′; siRRAD#2, 5′-GGACGGAGAAGAGGCAUCA UU-3′; siRRAD#3, 5′-AGGCAUCACUCAUGGUCUAUU-3′; and control siRNA (siC) were purchased from Dharmacon (Lafayette, CO). Cells (4 × 10^5^ cells per 60-mm dish) were transfected with 10 nM siRNAs using HiPerfect transfection reagents (Qiagen) according to the manufacturer’s instructions and were used for western blot analysis 48 h after transfection.

Full-length RRAD was cloned from HeLa mRNA for Flag-tagged cloning into pCMVTag2B (Clontech). Cells (4 × 105 cells per 60-mm dish) were transfected with 1 μg plasmid using Effectene transfection reagents (Qiagen) according to the manufacturer’s instructions and were used for western blot analysis 48 h after transfection.

The effective sequence (siRRAD#1, siRRAD#2) was cloned into the H1-shRNA vector (Genolution Pharmaceuticals Inc, Seoul, Korea). The sequence of nonsense shRNA against luciferase was provided by Genolution Pharmaceuticals Inc. shRNA constructs were transfected into cancer cells using Effectene transfection reagents (Qiagen) according to the manufacturer’s instructions. Stable transfectants were established in the presence of 100 μg/mL Zeocin selection.

### Cell growth assessment

To assess cell numbers, cells (1 × 10^5^ cells per 6-well plate, Corning Costar Corp, NY, USA) were transfected with siRNAs and incubated for 3 days. Adhered cells were trypsinized, stained with 0.2% trypan blue (CellTiter-Glo, Promega), and counted using a hemocytometer. Cell proliferation in each treatment was compared with that of untreated cells.

### Modified boyden chamber cell invasion assay

Cells (1 × 10^5^) were loaded into the top chamber of matrigel-precoated Transwell plates (8 mm pore size; Corning Costar). FBS (10%) was used as a chemoattractant in the bottom chamber. After incubation for 24 hours, cells in the bottom chamber were fixed and stained with 0.05% (w/v) crystal violet. The number of invading cells was quantified by counting those in five random fields of each membrane.

### Tube formation assay

*In vitro* angiogenesis was assessed using the Endothelial Tube Formation Assay Kit (CBA-200; Cell Biolabs, Inc., San Diego, CA, USA). Briefly, the Matrigel gel was thawed at 4 °C overnight and then bottom coated in a 96-well plate (50 μl per well) at 37 °C for 30 min. Next, 150 μl of media containing Human umbilical vein endothelial cells (HUVECs) (2 × 10^4^ cells) with siControl or siRRAD transfected cancer cell medium was added to each well on top of the solidified ECM gel and incubated at 37 °C for 18 h. Images were taken using an Axiovert 200 fluorescence microscope (Zeiss, Jena, Germany) at 40x magnification. Total tube length in each well was measured by the ImageJ program (National Institutes of Health, USA).

### ELISA assay

Secreted protein levels of VEGF and angiopoietin 2 were measured on culture media (200 μL) collected from siRRAD transfected cells. Protein levels of VEGF and angiopoietin 2 were measured using an ELISA kit for human VEGF and angiopoietin 2 (R&D Systems, Minneapolis, MN) according to the manufacturer’s instructions. A microtiter plate reader was used to read the plate at a wavelength of 450 nm.

### Glucose uptake and lactate production assay

For the analysis of glucose uptake, we employed the GluTracker glucose uptake assay kit (BioVision, K681, BioVision Inc., Milpitas, CA, USA) and carried out the experiment according to the manufacturer’s described. For each measurement, data from 10,000 single-cell events were collected using FACS verse (BD Bioscience).

To detect the lactate production from siRRAD transfected cells, lactate colorimetric assay was carried out to measure the total lactate content in the cell culture supernatant of the siRNAs transfected cells for 48 h using a Lactate Assay kit according to the manufacturer’s instructions (BioVision, K607).

### Annexin V assay

Cells (5 × 105 cells) were seeded in a 60-mm dish and incubated for 24 h and transfected with siRNAs. After incubation for 24 h, cells were treated with 5-FU (1 μg/ml) and incubated for 48 h. After washing twice with PBS, the cells were stained using the Annexin V-FITC/Propidium iodide apoptosis kit (BD Bioscience, San Jose, CA) according to the manufacturer’s instructions. Stained cells were detected and analyzed using FACS verse (BD Bioscience).

### Xenograft study and immunohistochemistry

Male BALB/c nude mice, 4–6 weeks old, were obtained from Orient Bio Inc (Seongnam, Korea). Mice were subcutaneously implanted with shRRAD expressed MKN1 (1 × 10^7^) or SW48 (5 × 10^6^) cells in 100 μl volume. The mice were randomized, and 5-FU treatment was started at 7 days after inoculation. 5-FU (1 mg/kg, intraperitoneal injection) were given twice per week. Tumor growth was measured using a digital caliper (Proinsa, Vitoria, Spain) every 3–4 days, and average tumor volumes were calculated using the following formula: V = (L × W^2^)/2, where V = volume (in cubic millimeters), L = length (in millimeters), and W = width (in millimeters). The mice were sacrificed, and tumors (three tumors per treatment group) were resected and frozen in liquid nitrogen until later use for western blot analyses. All mice experiments were conducted in accordance with the Institute for Laboratory Animal Research Guide for the Care and Use of Laboratory Animals, and the protocols were approved by the appropriate Institutional Review Boards at Samsung Medical Center (Agreement- 20141211001).

Immunohistochemical studies were performed with 4-µm-thick tissue sections using a PCNA (sc-56, Santa Cruz Biotechnology), CD31 (98941S, Cell signaling technology) and RRAD (ab75100, Abcam) antibody. Tissue sections were deparaffinized and subsequently rehydrated. Immunostaining was performed using a EnVision Detection System (DAKO, DAKO, Denmark) In brief, antigen retrieval was performed by heating the samples at 97 °C for 20 min in pH 6.0 HIER citrate buffer, blocking endogenous peroxidase activity with 3% hydrogen peroxidase for 5 min, and incubating the samples in a 1:100 dilution of the primary antibody for overnight at 4 °C.

### Human cancer tissue and PDCs

Thirty-nine matched pairs of primary CRC and normal colorectal tissue and PDCs (N = 11) and 45 matched pairs of primary GC and normal gastric tissue and PDCs (N = 48) were collected at Samsung Medical Center. All CRC and GC tumors and control tissues were confirmed by the hospital’s clinical pathology department. Ascites-derived cells from patients with metastatic CRC or GC with malignant effusion who were enrolled in the SMC Oncology Biomarker study (NCT#01831609) were screened for RRAD expression by western blot and real-time PCR.

All patients provided written informed consent. This study was performed in accordance with the Declaration of Helsinki and was approved by the Institutional Review Board of Samsung Medical Center.

### Statistical analysis

Data in the graphs represent mean ± SD of values from at least three independent measurements. To determine the differences in mean values, a two-tailed *t*-test was applied. Intergroup comparisons were made with paired two samples *t*-test. Differences were considered significant at P < 0.05.

### Ethics approval and informed consent

All patients provided written informed consent. This study was performed in accordance with the Declaration of Helsinki and was approved by the Institutional Review Board of Samsung Medical Center.

## Supplementary information

Supplementary figures

## Data Availability

All analyzed data from this study are included in this published article and its Supplementary Information. All data generated during the current study are available from the corresponding author on reasonable request.
