# Supplementary material for: RRAD expression in gastric and colorectal cancer with peritoneal carcinomatosis
Source: Sci Rep. 2019 Dec 19;9:19439. doi: 10.1038/s41598-019-55767-7 (PMC6923381; doi:10.1038/s41598-019-55767-7)
Supplement: Supplementary file 1 — Supplementary figures [file 41598_2019_55767_MOESM1_ESM.pdf]

RRAD expression in gastric and colorectal cancer with peritoneal carcinomatosis

Hee Kyung Kim[1,4*], Inkyoung Lee[2*], Seung Tae Kim[1], Jeeyun Lee[1], Kyoung-Mee Kim[3], Joon Oh Park[1], and Won Ki Kang[1]

[1]Division of Hematology-Oncology, Departments of Internal Medicine, Samsung Medical Center, Sungkyunkwan University School of Medicine, Seoul, Korea

[2]Biological Research Institute, Samsung Medical Center, Sungkyunkwan University School of Medicine, Seoul, Korea

[3]Department of Pathology, Samsung Medical Center, Sungkyunkwan University School of Medicine, Seoul, Korea

[4] Department of Internal Medicine, Chungbuk National University Hospital, Chungbuk National University College of Medicine, Cheongju, Korea

*These authors contributed equally to this work.

Corresponding author: Won Ki Kang, MD

Division of Hematology-Oncology, Department of Internal Medicine, Samsung Medical Center, Sungkyunkwan University School of Medicine, 81 Irwon-ro, Gangnam-gu, Seoul 06351, Korea

Tel: +82-2-3410-3451, Fax: +82-2-3410-1754, E-mail: wkkang@skku.edu

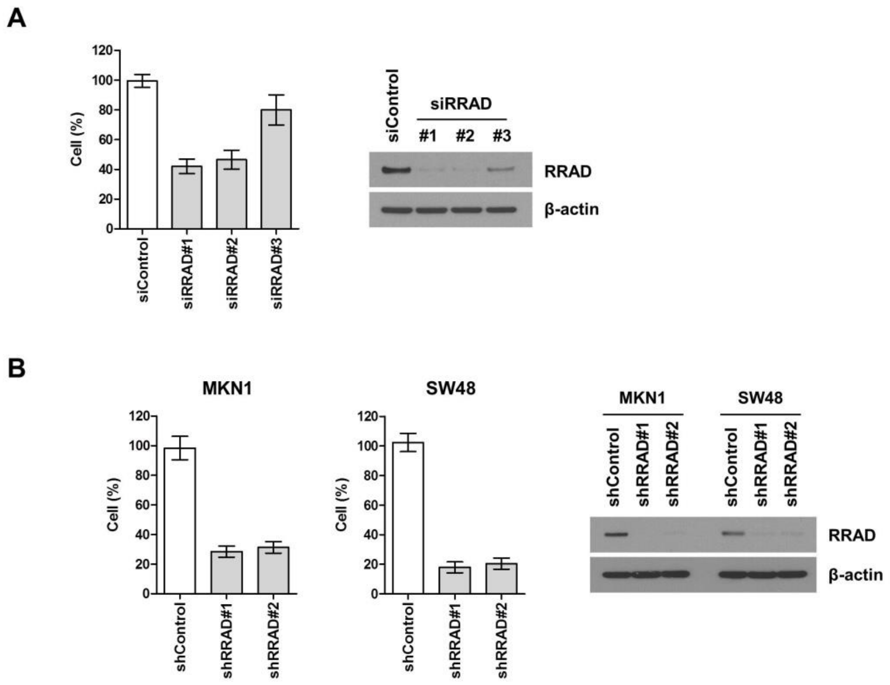

Supplementary Figure S1: **RRAD knockdown using 3 siRNAs and 2 shRNAs induced cell growth retardation.** The efficiency of RRAD knockdown was tested by immunoblotting.

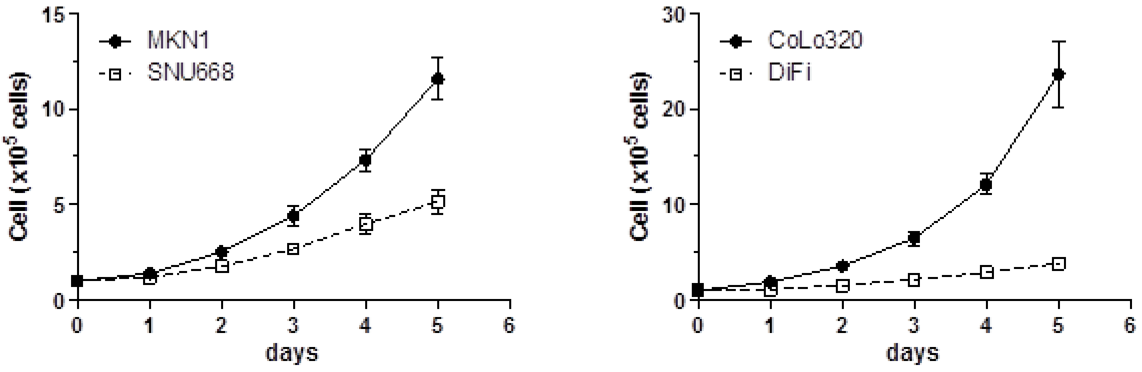

Supplementary Figure S2: RRAD-positive cell lines (strong RRAD expression by western blot, MKN1 and CoLo320) demonstrated faster growth rates compared to RRAD-negative (low or no expression of RRAD by western blot, SNU668 and DiFi).

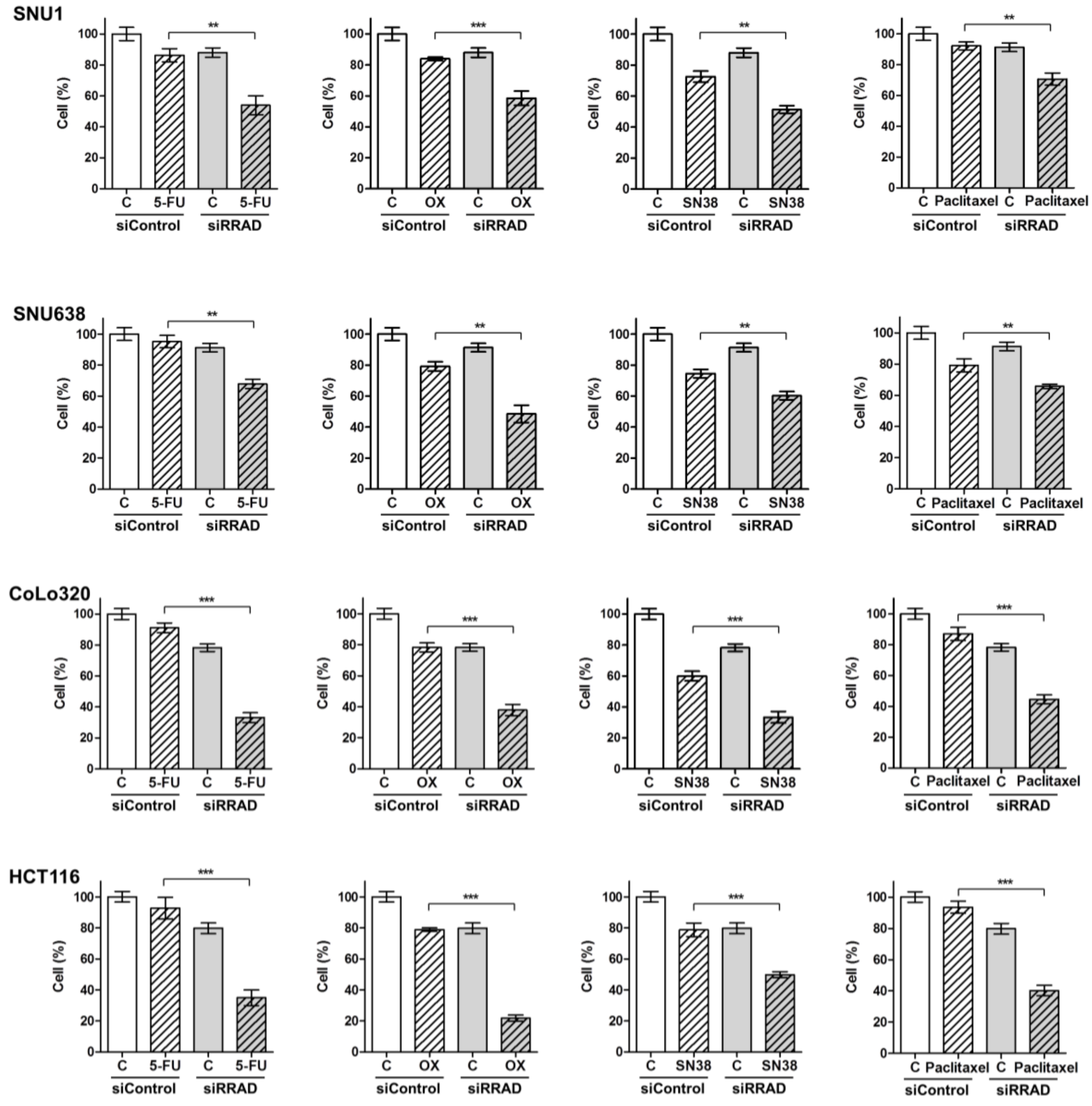

Supplementary Figure S3: Synergistic effects of RRAD inhibition with chemotherapy in other gastric cancer cell lines (SNU1, SNU638) and colorectal cancer cell lines (CoLo320, NCI-H716)

Significant decrease in cell proliferation with combination therapy was observed in all cell lines.

*P < 0.05, ** P < 0.01, *** P < 0.001.

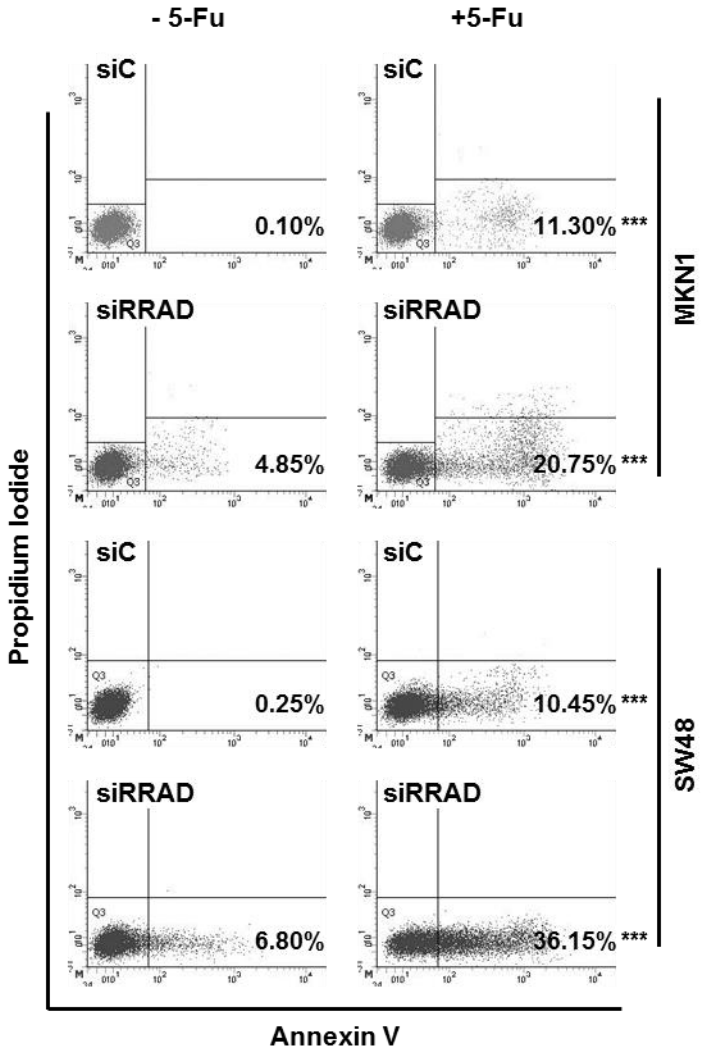

Supplementary Figure S4: Annexin V staining assay was used to assess the synergistic effects of RRAD inhibition and 5-FU.

Apoptosis was measured in Control and RRAD siRNA-transfected and 5-FU (1 μg/ml). Values represent mean ± SD of three independent experiments performed in triplicate; ***p<0.01.

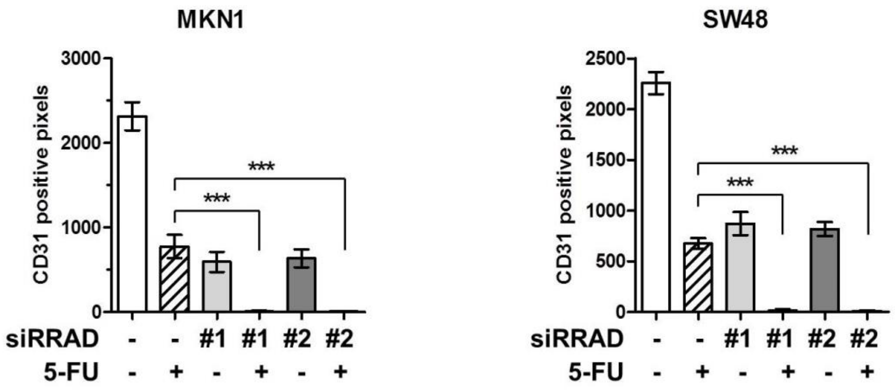

Supplementary Figure S5: Quantification of CD31-positive pixels

Combination therapy with 5-FU and siRRAD significantly reduced the expression CD31.

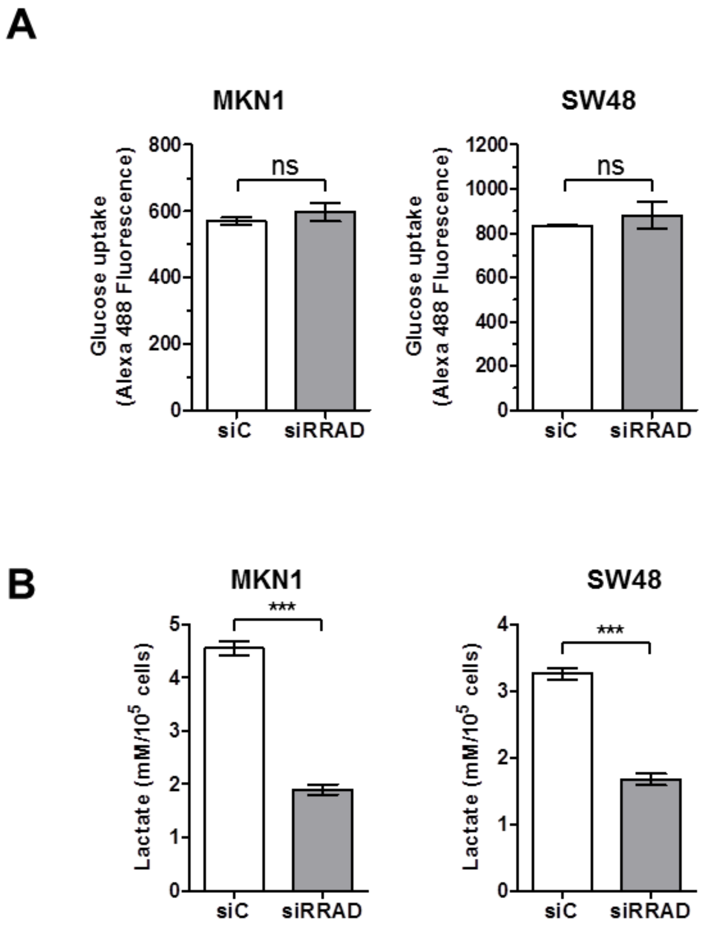

\

Supplementary Figure S6: Effect of RRAD knockdown on Glucose uptake and lactate production in vitro.

(A) Control and RRAD siRNA-transfected cells showed no difference in Glucose uptake.

(B) Lactate colorimetric assay detected a decreased level of lactate in the supernatants of siRRAD transfected cells compared to siControl transfected cells.

Fig.1A

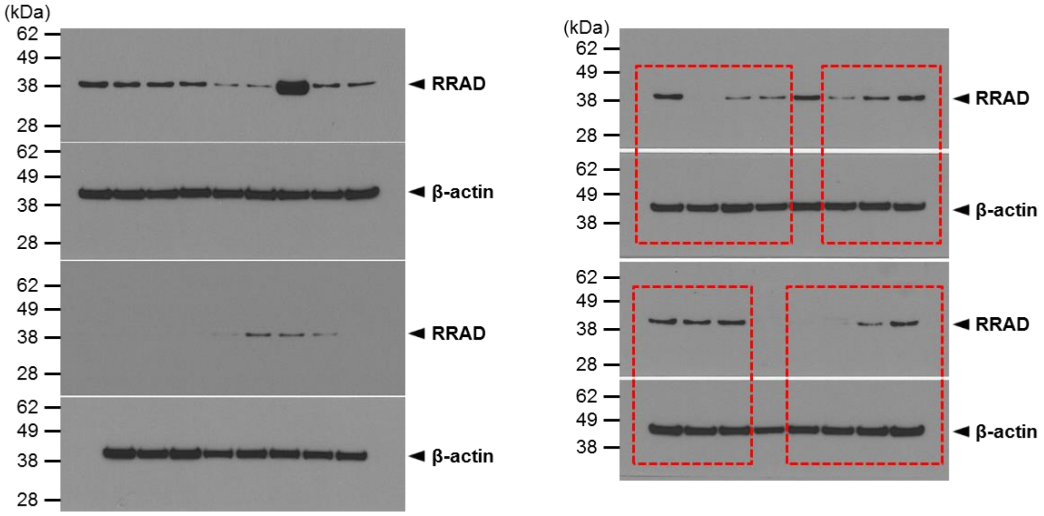

Fig.1B

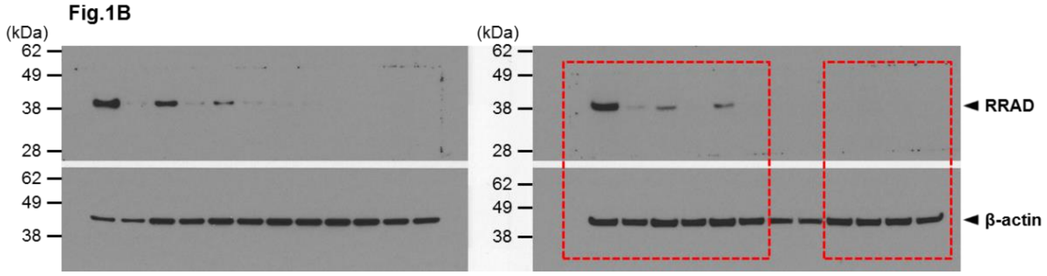

Fig.3A

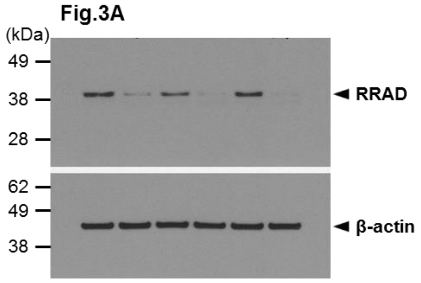

Fig.3B

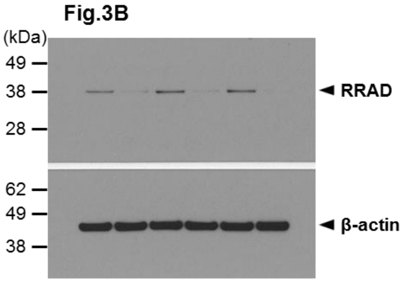

Fig.4C

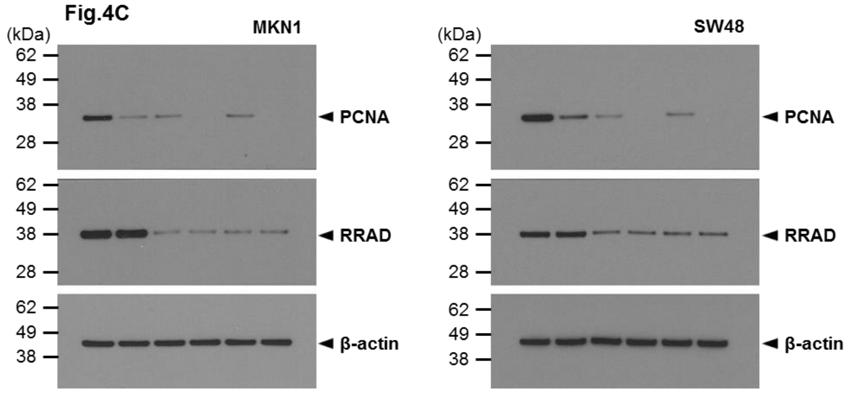

Supplementary Figure S7: Full-length blots corresponding to Figures 1, 3, 4

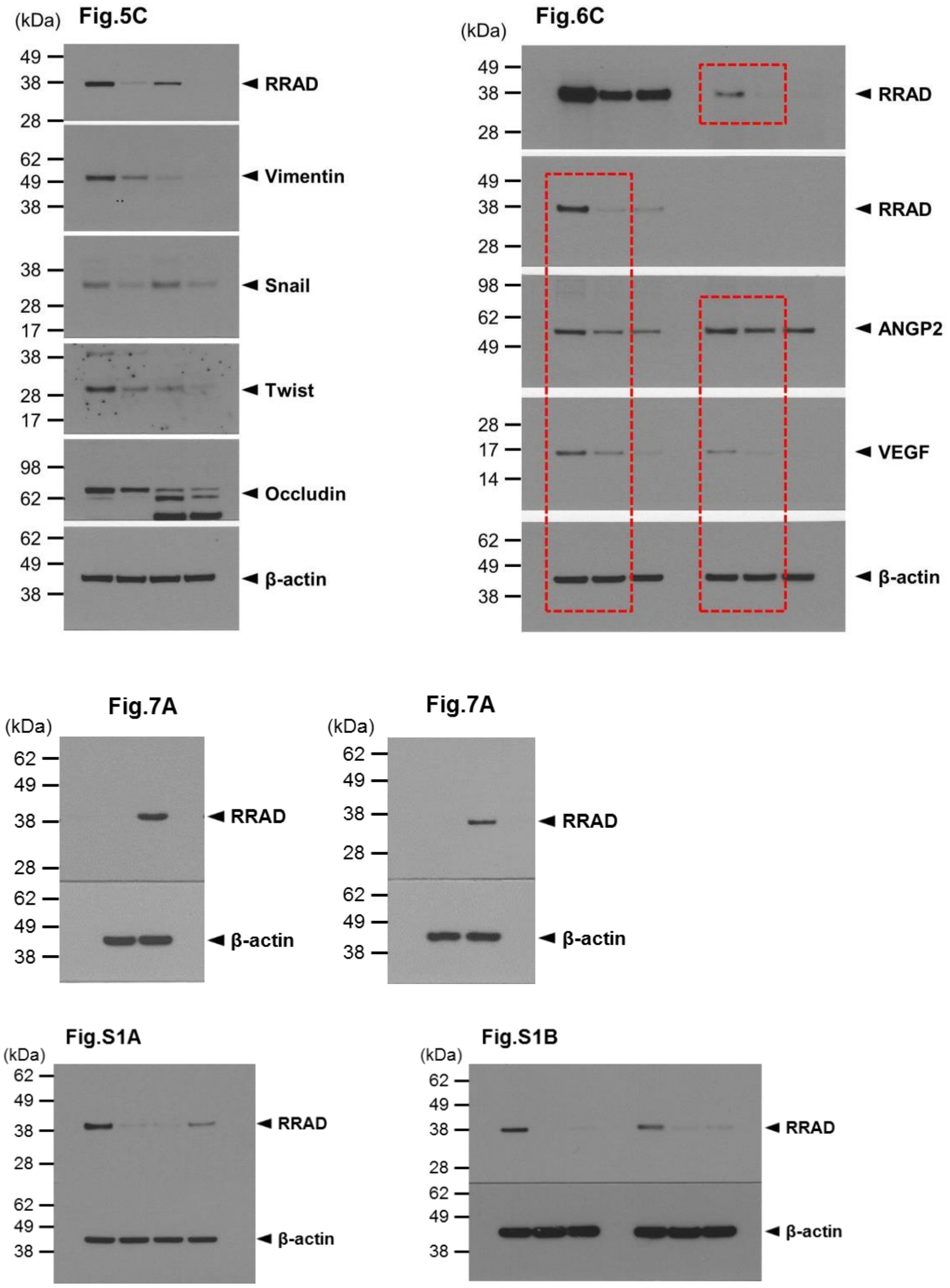

Supplementary Figure S8: Full-length blots corresponding to Figures 5,6,7 and S1.
